# Reflections on the upsurge of virtual cancer conferences during the COVID-19 pandemic

**DOI:** 10.1038/s41416-020-1000-x

**Published:** 2020-07-23

**Authors:** Valerie Speirs

**Affiliations:** Institute of Medical Sciences, School of Medicine, Medical Sciences and Nutrition, University of Aberdeen, Foresterhill, Aberdeen, AB25 2ZD Scotland UK

**Keywords:** Conferences and meetings, Cancer

## Abstract

The COVID-19 pandemic resulted in the cancellation or postponement of traditional face-to-face scientific conferences, necessitating a rapid change in the way new discoveries in cancer were shared with the cancer research community. Here I present personal reflections on the upsurge of virtual cancer conferences, discussing their pros and cons in the context of traditional face-to-face deliveries.

## Main

Springtime usually heralds the start of the conference season for academics, and many of us look forward to visiting different destinations to engage with colleagues from across the world to learn about the latest findings in cancer research, spend time discussing these in social settings and forging new collaborations. This year has been different; the identification of a cluster of pneumonia cases in Wuhan, China, in December 2019, caused by a novel coronavirus (2019-nCoV),^1^ resulted in the rapid escalation of the COVID-19 pandemic. Consequently, national and international travel was suspended, leading to the cancellation or postponement of traditional face-to-face scientific conferences, necessitating a swift change in the way discoveries in cancer research were delivered. Technological innovations in the digital space were embraced, offering the opportunity to provide conferences ‘virtually’, with delegates participating from their own homes.

One of the first cancer conferences to be affected by the pandemic was the American Association for Cancer Research (AACR) annual conference, scheduled to take place on 24–29th April in San Diego, California. A stalwart in the cancer research calendar, this was initially rescheduled for August 2020 as a traditional face-to-face conference, but the emerging pandemic and associated public health risk resulted in the organisers moving swiftly to replace this with a two-part virtual conference. The first part, AACR I, was delivered on 27–28th April, partly coinciding with the dates of the original conference, with AACR II scheduled for late June. Although the pandemic has brought many aspects of daily life to a standstill, people continue to receive cancer diagnoses and require treatment, and it was fitting that the AACR I plenary session was dedicated to discussing how the COVID-19 pandemic had impacted on the treatment and care of cancer patients, featuring speakers from areas that had been, at the time of the conference, most badly affected by the pandemic, including Wuhan, China; Milan, Italy; Madrid, Spain.

AACR I focused predominantly on clinical trials and drug discovery, providing a mechanism for the cancer research community to report the results of clinical trials that may benefit patient outcomes, avoiding unnecessary delays in reporting these as a result of the pandemic. This was realised with the efficacy and safety data of a MET inhibitor, capmatinib (Tabrecta), in treating non-small-cell lung cancer patients with METex14 skipping mutations, reported in the lung cancer clinical plenary session (https://www.abstractsonline.com/pp8/#!/9045/presentation/10786), resulting in its recent approval from the Food and Drug Administration (FDA).

The overall content of the AACR I virtual conference was broadly in line with what we have come to expect from a regular face-to-face conference, including oral and poster presentations, and was the trailblazer for virtual cancer meetings, attracting over 61,000 registrants from 140 countries (https://www.abstractsonline.com/pp8/#!/9045/presentation/10786). Others followed. In May, close to 10,000 delegates joined the European Society for Medical Oncology (ESMO) Virtual Breast Cancer Meeting, with all abstracts originally accepted for presentation at the face-to-face meeting remaining in the virtual programme. This multidisciplinary meeting brought together scientists and clinicians engaged in breast cancer research, and like AACR I, the keynote lecture focused on COVID-19, specifically how this had impacted on breast cancer treatment and clinical trials. The American Society of Clinical Oncology (ASCO) annual conference also went virtual with clinical trials given priority. In Europe, the 2020 Congress of the European Association for Cancer Research (EACR), originally scheduled in Torino, Italy, was replaced with a virtual event. However, not all cancer conferences have gone virtual. The National Cancer Research Institute (NCRI) annual conference, a 3-day event that provides a forum to showcase the latest advances in cancer research in the United Kingdom, was cancelled. Although not being offered in a fully virtual format, the NCRI is providing a series of weekly webinars to keep researchers up to date with ongoing developments in cancer research.

So far, virtual conferences have been free to attend, requiring creation of an online account and password to gain access. This contrasts the often-expensive conference registration fees plus associated travel and accommodation costs that delegates need to meet from their research budgets. This might explain the impressive numbers of delegates achieved, e.g., by AACR I and ESMO Breast Cancer, which far exceed the numbers of delegates typically attending the equivalent face-to-face event. Whether virtual events will continue to be free in the long term remains to be seen, as there are still costs associated with running these, and dedicated event management companies are now offering virtual event platforms. Aside from financial benefits to delegates, virtual conferences are attractive to those with restricted ability for international travel, e.g., those with young children or other caring responsibilities. Virtual conferences have also increased international reach to oncology stakeholders, allowing meeting registrants from across the globe to participate, either via live streaming or on demand to accommodate those in different time zones.

Like traditional conferences, virtual conferences require considerable effort to put together; it is not a case of simply putting together a few pre-recorded sessions, and some may be better resourced than others in their ability to deliver this. There have been some technical issues with IT and variability in the delivery of presentations, and even within conferences, some sessions have been hybrids of live delivery and pre-recorded presentations. In the case of the former, streaming has sometimes been an issue, although this may result from personal Internet connectivity, beyond the control of the conference organisers. There were often unexpected delays between sessions, a time when one would normally be moving to another session held in an adjoining hall or using the time for networking or refreshment breaks. Session moderators were not always visible, and the ability to ask questions ‘off the cuff' was lost with questions having to be typed into chat boxes with the moderator selecting which ones to ask, hence losing some of the spontaneity, reaction to body language and challenge associated with fielding face-to-face questions. However, we are still learning the best ways to navigate this new virtual conference world. Best-practice guidelines have recently been published to facilitate optimal scheduling and delivery of virtual conferences.^2^

Most virtual cancer conferences staged thus far have included all the features we have come to expect from face-to-face conferences, including satellite symposia, virtual exhibitions and pre-recorded poster discussion sessions, delivered as short online presentations and late-breaking work. However, traditional poster sessions are, arguably, the biggest casualties of virtual conferences. Although we have become used to electronic poster boards at face-to-face conferences, the presenter of the poster is usually there to explain their work. Virtual poster sessions are not the same; with no one around to talk you through the work, the interactive nature is lost. For early career researchers, the apprehension (and excitement) we have all experienced of presenting our first poster at a conference and the prospect of having to field questions from experts in the field cannot be replicated in a virtual world.

Input from patient advocates is also lost. These have been a welcome inclusion at cancer conferences in recent years, with their personal views and experiences injecting fresh perspectives into the cancer space, introducing a more patient-centric approach to clinical trials and, increasingly, shaping laboratory research.^3^ Most major cancer conferences now offer advocate bursaries giving recipients the opportunity to network with fellow advocates, clinicians and scientists allowing bidirectional learning. This has been a very successful formula, but something that is unlikely to be sustained in virtual settings, where the patient voice may not be heard. If virtual conferences are to continue, this must be addressed.

For delegates, valuable networking opportunities are lost too, alongside the opportunity to turn to your neighbour in the audience to muse over a point made by a speaker spontaneously and catch up with colleagues and friends from overseas or strike up a conversation at a poster session. While some events have tried to address this, offering virtual networking using secure online chats or one-on-one video calls, these were not the same as meeting in person. From a personal perspective, attending AACR, ASCO, ESMO etc. from the home environment was a very different experience with the conference vibe missing.

Is this the future for cancer conferences? In some respects, the upsurge of virtual conferences is analogous to the explosion of MOOCs (massive open online courses) in the early 2010s. MOOCs became very popular in a short space of time, and were seen as a mechanism of using the Internet to bring a snapshot of higher education to those who may not otherwise have had this opportunity.^4^ At one stage, MOOCs were perceived as the future of higher education, but with sustainability questioned,^5^ just as quickly as they became popular, their popularity waned. Once the pandemic eases and travel restrictions are relaxed, the same may apply to virtual conferences. It is still early days, so time will tell.

## Data Availability

Not applicable.
